# Baculovirus Nuclear Import: Open, Nuclear Pore Complex (NPC) Sesame

**DOI:** 10.3390/v5071885

**Published:** 2013-07-23

**Authors:** Shelly Au, Wei Wu, Nelly Panté

**Affiliations:** Department of Zoology, University of British Columbia, 6270 University Boulevard, Vancouver, British Columbia V6T 1Z4, Canada; E-Mails: shellyau@hotmail.com (S.A.); wwlyl1985@hotmail.com (W.W.)

**Keywords:** baculovirus, AcMNPV, nuclear import, nuclear pore complex, viruses

## Abstract

Baculoviruses are one of the largest viruses that replicate in the nucleus of their host cells. During infection, the rod-shape, 250-nm long nucleocapsid delivers its genome into the nucleus. Electron microscopy evidence suggests that baculoviruses, specifically the Alphabaculoviruses (nucleopolyhedroviruses) and the Betabaculoviruses (granuloviruses), have evolved two very distinct modes for doing this. Here we review historical and current experimental results of baculovirus nuclear import studies, with an emphasis on electron microscopy studies employing the prototypical baculovirus *Autographa californica* multiple nucleopolyhedrovirus infecting cultured cells. We also discuss the implications of recent studies towards theories of nuclear transport mechanisms.

## 1. Introduction

Baculoviruses are a large and diverse group of rod-shaped, enveloped, double-stranded DNA viruses that replicate in the nucleus of their host cells. They are pathogenic to arthropods, mainly insects, and are ubiquitously found in the environment. Members of the *Baculoviridae* family have been isolated from more than 700 host species. Baculoviruses play a role in the control of natural insect populations, and have long been used as bio-insecticides to control insect pests in agriculture and forestry (reviewed in [1,2]). In biomedical research, baculoviruses are better known as potent protein expression vectors (reviewed in [3,4,5,6]), and have even been used in the production of vaccines (reviewed in [7]). Moreover, because of its ability to transduce mammalian cells without viral replication, baculovirus has been proposed as a possible new class of gene therapy vector (reviewed in [8,9,10,11,12]). Because of the obvious importance of baculoviruses, they have been studied to a great extent; in particular their prototype *Autographa californica* multiple nucleopolyhedrovirus (AcMNPV). Even so, many steps of the baculoviral life cycle remain poorly characterized. One of these is the mechanism by which baculovirus delivers its genome into the nucleus of the host cell. As nuclear import is a vital event in the baculovirus life cycle, understanding this mechanism will help to enhance the use of baculovirus as effective insecticide, protein expression system and more efficient vector for gene therapy and vaccine production. 

This review highlights our current knowledge of baculovirus nuclear import. We first briefly introduce the taxonomy of the family *Baculoviridae*, the structures of the baculovirus’s two distinct viral phenotypes, and briefly summarize the infection cycle of one of these phenotypes, the budded virus, in cultured cells, with emphasis in the steps before nuclear import. We then discuss earlier and more recent studies that elucidate the mechanism of baculovirus nuclear import. Finally, we discuss how these studies pertain to various models for the mechanism of nuclear transport.

## 2. Baculovirus: Classification, Structure and Life Cycle

### 2.1. Baculovirus and Its Classification

Most baculovirus isolates have been made from diseased caterpillars (Lepidoptera); some have been from sick sawflies (Hymenoptera) and a very few from infected mosquito larvae (Diptera). Baculoviruses are recognized in their diseased hosts by the large proteinaceous bodies called occlusion bodies (OBs) they produce. Based on the distinct OB morphology, the family *Baculoviridae* was historically divided into two major genera: nucleopolyhedrovirus (NPV) and granulovirus (GV). NPV nucleocapsids are enclosed either singly (SNPV) or multiply (MNPV) within an envelope and embedded within a crystalline matrix of the protein polyhedrin, forming large (0.15–15 µm) polyhedral OBs. GVs contain a single enveloped nucleocapsid embedded within the protein granulin into a small (0.13 × 0.5 µm) oval-shaped OB (reviewed in [13]). 

The advent of molecular technology enabled baculovirus classification to take a leap forward. By adding genome sequence information to existing morphological descriptive data, a better understanding of the evolutionary relatedness among the baculoviruses was obtained. Not surprisingly, the viral sequence data showed distinct clusters that co-aligned with the taxonomy of the hosts. This implied that viruses with lepidopteran hosts, for example, were more closely related to each other than they were to the viruses infecting dipteran or hymenopteran hosts, and vice versa. This observation is especially interesting when considering that the Lepidoptera are the most recent order of insects to have appeared (~232 mya), while the Diptera and Hymenoptera are older (~260 mya and 309 mya, respectively). Viruses isolated from lepidopteran hosts include GVs, MNPVs and SNPVs, while viruses from the two older orders are so far limited to SNPVs. 

**Figure 1:**
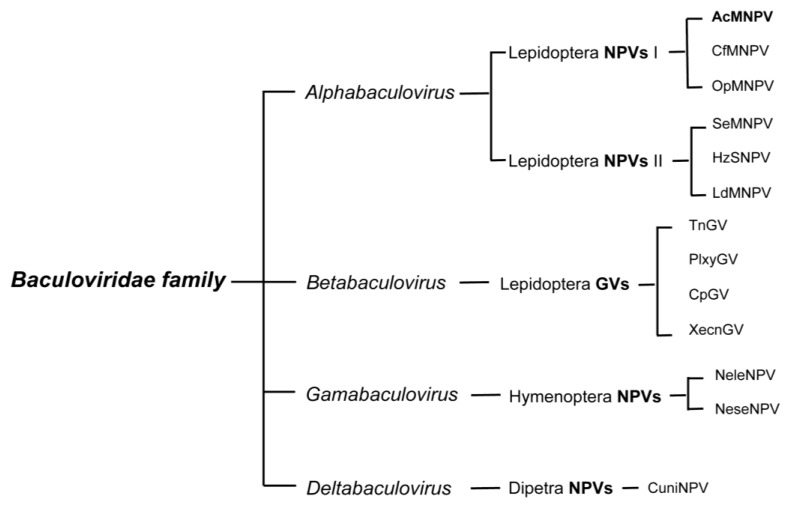
Classification of the *Baculoviridae* family. Only a small subset of characterized species within each group is listed. AcMNPV, the most studied baculovirus and most commonly used viral vector for baculovirus expression vector systems, belongs to the type I Alphabaculovirus genus.

Genomic sequences of GVs and NPVs that infect Lepidoptera form two distinct clusters and represent the genera of Alpha- and Betabaculoviruses, respectively. Viruses isolated from saw flies constitute the Gammabaculovirus genus and those isolated from mosquito larvae are the Deltabaculoviruses (Figure 1) (reviewed in [14,15]). The Alphabaculoviruses are further divided into Group I and II; the two groups differ in gene content, but most noticeably in the fusion protein encoded by each group. Group I NPVs, such as the archetype AcMNPV, use a GP64 fusion protein, and Group II NPVs use an F-protein [16]*.*

Baculoviruses are normally named for the host from which they were first isolated and the type of OB formed. Thus, for example, the baculovirus that infects the alfalfa looper *Autographa californica* is named AcMNPV, and that from the spruce budworm *Choristoneura fumiferana* is named CfMNPV.

### 2.2. Baculovirus Structure

**Figure 2:**
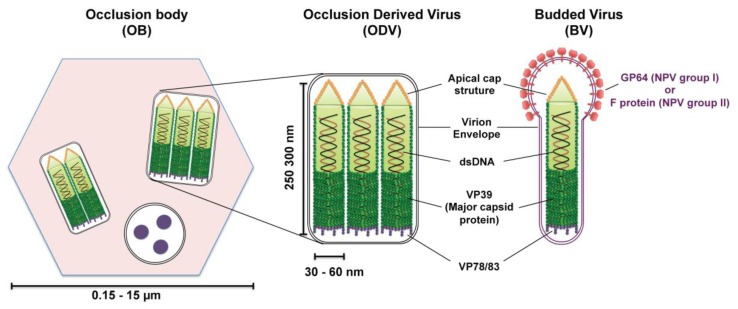
Schematic diagrams of the structure of baculovirus occlusion bodies (OB), occlusion-derived virion (ODV) and budded virion (BV). ODVs are embedded in a crystalline matrix of protein to form OBs. Shown here is the OB of nucleopolyhedroviruses (NPVs) (the vesicle containing three round structures is a cross section of an ODV). The ODV and BV envelope and their nucleocapsid(s) contain numerous proteins; only the proteins discussed in this review are outlined in the diagram. The major nucleocapsid protein VP39 constitutes the barrel of the nucleocapsid and is present in the whole nucleocapsid. VP78/83 is located at one end of the nucleocapsid, presumably at the blunt end. The fusion protein GP64 (NPV group I) or F protein (NPV group II) is found throughout the BV envelope but forms peplomers on one end, presumably the conical end.

Most baculoviruses produce two types of infectious viral particles: the budded virion (BV) and the occlusion-derived virion (ODV) (Figure 2). While all four genera of baculoviruses form ODVs, only Alpha-, Beta-, and Deltabaculoviruses generate BVs [14]. The two forms of virions are produced at different times and subcellular places during the replication cycle. BVs are produced during the initial replication when nucleocapsids bud from infected cells and obtain their envelopes from the plasma membrane. Thus, BV contains a single nucleocapsid and a plasma membrane-derived envelope, which contains the viral fusion proteins (GP64 or F protein). ODVs are produced during the very late phase of replication and are formed in the nucleus by envelopement of a single or multiple nucleocapsids per virion, which then become incorporated within the protein matrix (polyhedrin for NPVs or granulin for GVs) forming OBs that are released into the environment upon death of the infected larva. While ODV is involved in virus transmission between insect larvae by infecting midgut cells of feeding caterpillars, BV is the infectious form responsible for cell-to-cell transmission within the host and in cell culture (reviewed in [17]).

The baculovirus nucleocapsid, which is the central component of both virion phenotypes, has a rod‑shaped morphology with two distinct ends: apical cap end with a small protuberance and one end blunt (Figure 2). The size of the nucleocapsid varies, ranging from 250–300 nm in length by 30–60 nm in diameter [13]. This nucleocapsid encloses a circular, super-coiled, double-stranded DNA genome of 80–180 kbp that encodes between 90–180 genes (reviewed in [13,17]). The protein composition of ODV and BV nucleocapsids are similar but distinct from each other, with ten similar proteins found in both nucleocapsids and half a dozen of proteins that are specific to each nucleocapsid [18,19,20]. Only the proteins pertinent to this nuclear import review will be mentioned here. The major nucleocapsid protein, VP39, is a 39-kDa protein that constitutes the barrel of the nucleocapsid (reviewed in [17]). VP78/83 located at one end of the nucleocapsid is involved in actin nucleation during viral infection (reviewed in [17]). 

### 2.3. Budded Virus Infection Cycle

**Figure 3:**
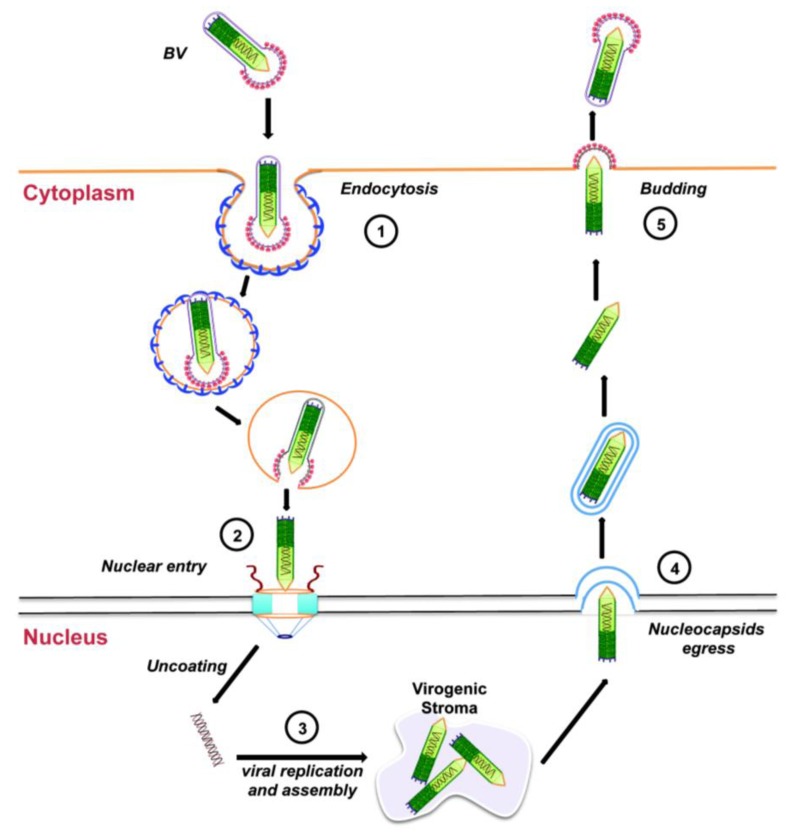
Budded virus infection cycle in cultured cells. See text for details.

Entry of the BV form of AcMNPV into tissue-cultured cells is through endocytosis (Figure 3, step 1). Upon acidification of the endosome, the endosomal membrane fuses with the viral envelope, resulting in the release of the nucleocapsid into the cytoplasm, which is then transported towards the nucleus using actin polymerization (Figure 3, step 2; see Section 3.2 below). Next, the viral DNA is released into the nucleus and viral replication begins with the formation of the virogenic stroma near the center of the nuclei of infected cells (Figure 3, step 3). This is the site of transcription and replication of viral DNA, and nucleocapsid assembly. During the early phase of replication (from 0 through 6 to 9 h after infection), genes essential for viral DNA replication are transcribed. DNA replication, followed by nucleocapsid assembly, occurs during the late phase of replication (between 6 to 18 h after infection). Progeny nucleocapsids leave the nucleus (Figure 3, step 4), acquiring an envelope from the nuclear membranes that is eventually lost. These nucleocapsids finally travel to the plasma membrane where they bud off acquiring a glycoprotein-rich envelope from the cell membrane to generate BVs (Figure 3, step 5). These BVs cause systemic infection by infecting neighboring cells and the infectious cycle begins again. 

## 3. Baculovirus: Cellular Events before Nuclear Import

### 3.1. Cellular Entry of Budded Virus

BVs contain envelope glycoproteins, such as GP64 for Group I of Alphabaculovirus, which are important for cell binding [21,22] and efficient cell surface budding of the virus [21,23]. The cellular receptors for these viral glycoproteins are currently unknown. In mammalian cells, BV cell entry has been shown to occur through clathrin-dependent endocytosis [24,25], as well as by other endocytic pathways, including clathrin-independent [26], direct fusion with the plasma membrane at low-pH [27,28], and macropinocytosis [29]. Fusion of the viral envelope with the endosomal membrane is also mediated by GP64 [27].

### 3.2. Intracellular Trafficking towards the Nucleus

A common theme for viruses is to use microtubules for cytoplasmic trafficking of their nucleocapsids towards the nucleus, whereas they could use microtubules or actin for egress of progeny nucleocapsids (reviewed in [30,31,32]). However, baculovirus uniquely uses actin for cytoplasmic transport of its nucleocapsid towards the nucleus [33,34]. Once baculoviral nucleocapsids are released into the cytoplasm, thick actin cables can be detected 1–4 h after infection [35,36]. These actin tails originate from the VP78/83 protein located at one end of the nucleocapsid [34,37]. VP78/83 is a viral Wiskott-Aldrich syndrome protein (WASP)-like protein that acts as an actin nucleation-promoting factor, essential for baculovirus infection [38,39]. Treating baculovirus infected cells with actin depolymerizing drugs slows baculoviral infection, and treatment with Arp2/3 inhibitors results in a decrease in the velocity of nucleocapsid movement toward the nucleus [34,40]. In addition, mutations in the Arp2/3 binding region of VP78/83 (Ile358 to Ala358) cause partial defects in actin polymerization and a reduced velocity of nucleocapsid movement toward the nucleus [34,41].

## 4. Nuclear Import of the Baculovirus Nucleocapsid

Baculovirus replicates in the nucleus of the infected cell, therefore, its viral genome must enter the host nucleus. Although cellular nuclear import occurs through the nuclear pore complex (NPC) and uses nuclear localization signals (NLSs) and nuclear import receptors (importin or karyopherin), an emerging picture from this field is that different viruses have developed different strategies to deliver their genome into the nucleus (reviewed in [42]). These include mechanisms such as transport of the intact nucleocapsid through the NPC, ejection of the nucleic acid through the NPC leaving the intact empty (devoid of DNA) capsid at the cytoplasmic side of the NPC, capsid disassembly at the NPC followed by transport of the genetic material through the NPC, and even nuclear entry through the nuclear membranes without using the NPC. In principle, baculovirus could use any of these mechanisms to deliver its DNA into the nucleus. Unfortunately, nuclear import of baculovirus is an understudied topic and both viral and cellular proteins involved in this process are currently unknown. 

Even less is known about the nuclear import of baculovirus proteins. Several baculoviral proteins that are expressed earlier during infection regulate viral transcription and are essential for viral DNA replication in the nucleus. Thus, these proteins must be imported into the nucleus following their synthesis in the cytoplasm. From the several baculoviral proteins that must enter the nucleus during infection, only the nuclear import of the late expression factor 3 (LEF-3) has been characterized in some detail [43,44]. LEF-3 contains an NLS that mediates its nuclear import, but also the nuclear import of its interacting protein P143, which is a helicase essential for viral DNA replication. It is possible that viral proteins exposed on the capsid may mediate the nuclear import of the baculovirus nucleocapsid. From all known capsid proteins of the BV, only VP80 has been described to contain a putative classical NLS (amino acids 424–439) [45]. However, it remains to be demonstrated whether this is a true NLS and whether it mediates the nuclear import of the baculovirus nucleocapsid. 

### 4.1. Early Studies from the 1960s to 1980s

The first studies to address the nuclear import of baculovirus were performed by Summers, who infected the larvae of the cabbage looper *Trichoplusia ni* (Tn) with the Betabaculovirus TnGV and analyzed larvae’s midgut tissues by electron microscopy at different time post-infection [46,47]. These studies revealed TnGV nucleocapsids associated end-on with the cytoplasmic face of the NPC at 2 to 6 h after infection, but not inside the nucleus. Strikingly, empty capsids were also found docking at the cytoplasmic side of the NPC. Based on these results, it was suggested that the GV genome is released into the nucleus through the NPC [46]. A similar mechanism is now well established for herpes simplex virus, whose nucleocapsid attaches to the cytoplasmic side of the NPC and ejects its nucleic acid into the nucleus through the NPC, leaving an empty capsid at the NPC [48,49].

Similar to the studies by Summers [46,47], a subsequent study using an Alphabaculovirus reported intact nucleocapsids associated to the cytoplasmic face of NPCs, some of which were partially electron-dense [50]. This is an indication that upon nucleocapsid docking at NPCs, only the viral genome of Alphabaculovirus crosses the NPC leaving empty capsids behind; a nuclear import mechanism similar to that observed for Betabaculoviruses and herpes simplex virus. In support of this mechanism, a biochemical assay using tissue cultured insect cells (*Spodoptera frugiperda* or *Sf* cells) infected with the Alphabaculovirus TnMNPV detected quick uncoating of nucleocapsids in the cytoplasmic fractions of infected cells [51]. Since this is a biochemical approach, it is possible that capsids attached to the cytoplasmic face of the NPC may have detached yielding empty capsids in the cytoplasmic fractions. However, several additional electron microscopy studies using a number of different Alphabaculovirus NPVs and either inoculated larvae or infected tissue cultured insect cells demonstrated intact nucleocapsids and partially empty nucleocapsids inside the host cells’ nucleus at late stages of infection [52,53,54,55,56,57,58]. As the NPC can accommodate transport of cargo up to 39 nm in diameter [59], it is possible that intact baculovirus nucleocapsids with diameters of about 30–40 nm are small enough to pass lengthwise through the NPC. However, it is not clear whether Alphabaculovirus nucleocapsids found in the nucleus of host cells were imported from the cytoplasm or were newly synthesized nucleocapsids. 

From these earlier studies it was established that nuclear import of Betabaculoviruses involves ejection of the genome through the NPC leaving empty capsids at the cytoplasmic face of the NPC. In contrast, these studies suggest that the nuclear import of some Alphabaculovirus NPVs can use two different nuclear import mechanisms: one similar to Betabaculovirus GV, and a second one involving translocation of the intact nucleocapsid into the nucleus through the NPC. It remains to be determined which viral and cellular proteins/factors are involved in each mechanism, and under which conditions members of the genus Alphabaculovirus use the first or the second mechanism. It is also unclear how Alphabaculoviruses could have evolved two different mechanisms for viral genome delivery; perhaps this genus can be further reclassified based on their mode of viral genome release into the nucleus.

### 4.2. Recent Advances in our Understanding of Baculovirus Nuclear Import Using AcMNPV

As described above earlier studies indicated that Alphabaculoviruses have evolved a mechanism to deliver its intact nucleocapsid into the nucleus, presumably through the NPC. Although the experimental evidence for this mechanism was only the detection of either intact or partially empty nucleocapsids in the nucleus, these could have reached the nucleus by other ways than through NPCs. For example, they could be progeny nucleocapsids generated during infection, they could have reached the nucleus during mitosis when the nuclear envelope completely disassembles, or they could have enter the nucleus like parvoviruses which transiently disrupt the nuclear membranes and enter the nucleus through the resulting gaps (reviewed in [42,60]).

To solve this puzzle, non-dividing mammalian cells were used in recent studies. This eliminated the possibility that progeny nucleocapsids could be detected in the nucleus, as baculovirus does not replicate in mammalian cells. In addition, the nuclear envelope remains intact in non-dividing mammalian cells and therefore the possibility of breakages in the nuclear envelope to allow nucleocapsid entry into the nucleus would be invalid. Using non-dividing mammalian cells and electron microscopy, van Loo and colleagues found intact electron-dense AcMNPV nucleocapsids docked at the cytoplasmic face of NPCs and inside the nucleus of transduced cells 4 h post-infection, suggesting that nuclear import of the intact nucleocapsid occurs through the NPC [40]. That the nucleocapsid crosses the NPC was further supported in a more recent study using High Five insect cells infected with AcMNPV in the presence of a truncated importin-β [34]. Under normal cellular conditions, importin-β acts as a cellular receptor that facilitates nuclear import of large cargoes by binding to various proteins residing on the NPC, called nucleoporins; however the truncated importin-β blocks nuclear import through the NPC because it cannot dissociate from the nucleoporins [61]. Ohkawa and colleagues used fluorescence microscopy to visualize the ability of the truncated importin-β to block the nuclear transport of fluorescently-labeled AcMNPV nucleocapsids, and found that indeed nucleocapsids were excluded from the nucleus in the presence of the truncated importin-β; indicating that the NPC is the route of nuclear entry for AcMNPV nucleocapsids [34]. 

**Figure 4:**
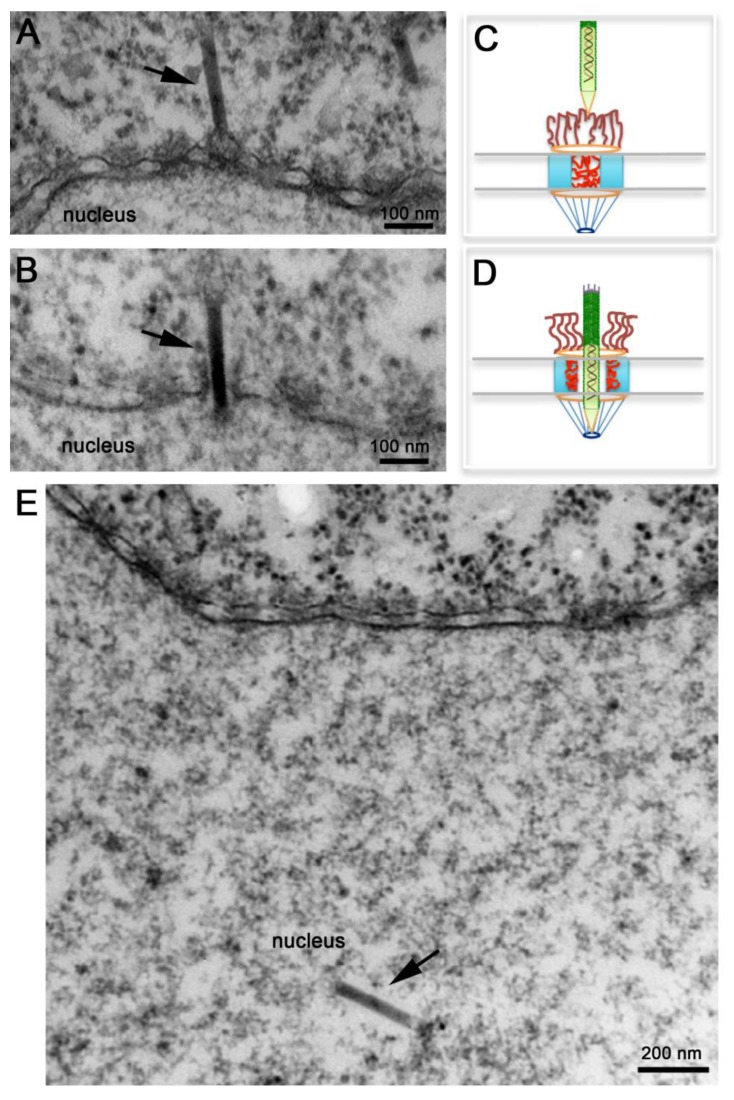
Nuclear import of AcMNPV Nucleocapsids. Electron micrographs of nuclear envelope cross-sections from *Xenopus* oocytes that have been microinjected with AcMNPV nucleocapsids and processed for embedding and thin-section electron microscopy. Intact nucleocapsids (arrows) were detected docked at the nuclear pore complex (NPC) prior to nuclear import (**A**), midway through the NPC (**B**), and inside the nucleus (**E**). (**C**, **D**) Schematic diagrams of NPCs illustrating “close” and “open” states respectively. In (**C**) NPC cytoplasmic filaments and nucleoporins within the central channel prevent the passage of the nucleocapsid. In (**D**) NPC cytoplasmic filaments straighten out, and nucleoporins in the central channel retract towards the body of the NPC to free up the passageway for the nucleocapsid to cross the NPC.

Several electron microscopy studies discussed above detected baculovirus nucleocapsids docked at the cytoplasmic side of the NPC or inside the nucleus, but never in the middle of the NPC. More recently, Au and Panté used microinjection of *Xenopus laevis* oocytes with purified AcMNPV nucleocapsids in combination with electron microscopy and electron tomography to demonstrate that indeed these nucleocapsids cross the NPC [62]. Microinjection of *Xenopus laevis* oocytes is a popular system to study nuclear transport of any substrate, including viral capsids [63,64], because of the ease to inject the large oocyte and because a mature oocyte contains a large number of NPC (5 × 10^7^ NPCs; [65]) densely packed. As illustrated in Figure 4A, oocytes microinjected with de-enveloped AcMNPV nucleocapsids yielded nucleocapsids docked at the cytoplasmic side of the NPC in what appear to be the NPC cytoplasmic filaments [62]. Notably, these nucleocapsids always had their apical end interacting with the NPC (e.g., this end enters the NPC first) (Figure 4A). In their studies Au and Panté [62] did not observed empty capsids, which are not electron-dense, docked at the NPC, ruling out a mechanism of nuclear import like the one used for Betabaculoviruses. At later times post-injection, AcMNPV nucleocapsids were found inside the nucleus of the oocyte (Figure 4E). As baculoviruses do not replicate in *Xenopus* oocytes, nucleocapsids found inside the nucleus must have been imported from the cytoplasm through the NPC. Strikingly, nucleocapsids were also found midway through the NPC (Figure 4B) demonstrating for the first time that the AcMNPV nucleocapsid crosses the NPC intact [62]. Transport of the intact nucleocapsid through the NPC was also demonstrated using conditions that block nuclear import, such as microinjecting the oocytes with the lectin wheat germ agglutinin, which binds to nucleoporins and essentially clogs the NPC inhibiting nuclear transport. Oocyte pre-injected with this lectin and then injected with AcMNPV nucleocapsids did not yield nucleocapsids in the nucleus [62], confirming previous findings by Ohkawa and colleagues that the AcMNPV nucleocapsid crosses the NPC intact [34]. These results rule out a mechanism of nuclear entry of baculovirus through the nuclear membrane as for parvovirus. It remains to be determined whether the intact nucleocapsid has functional NLSs and which cellular receptors (from the large importin or karyopherin family) facilitate the nuclear import of the nucleocapsid.

Transport of an intact AcMNPV nucleocapsid through the NPC was also confirmed by electron tomography, and a remarkable 3D image of a nucleocapsid in the midst of crossing the NPC was published [62]. Noticeable NPCs not engaged in nucleocapsid nuclear transport or those with nucleocapsids docked to their cytoplasmic filaments appear electron-dense (Figure 4A), as if nucleoporins residing within this gate prohibit the passage of the nucleocapsid (Figure 4C). Presumably cellular receptors or signaling events that are yet unknown function as the magical phrase ‘Open, Sesame’ that notifies nucleoporins in the central channel to move towards the body of the NPC unlocking this gate and leaving the NPC in an ‘open’ state that allows the nucleocapsid to get across it (Figure 4D). Moreover, such an unlocked mechanism seems to completely open up the NPC central gate so that an apparent space or a less electron-dense area of about 10 nm was detected around the nucleocapsid in electron micrographs and electron tomograms of nucleocapsids that were in the middle of NPCs [62]. Even the NPC cytoplasmic filaments, which usually have a kinky appearance in electron micrographs [66], appear straightened out (compare the short filaments emanating from the NPC into the cytoplasm in Figure 4A,B). It appears that the NPC cytoplasmic filaments and nucleoporins residing in the NPC central channel re-localize to clear out the entire passageway for the rod-shaped nucleocapsid to traverse the NPC and enter the nucleus (Figure 4D).

### 4.3. Using Baculovirus to Understand How the NPC Functions

Despite the obvious importance of nuclear transport and despite many recent advances in this field, many aspects of its molecular mechanism, its structural basis, or its regulation remain elusive. In particular, the precise molecular mechanism of translocation of molecules through the NPC remains to be elucidated. Nevertheless, several nuclear transport models (reviewed in [67]) have been proposed and are a major topic of debate and controversy in the field of nuclear transport. One such model called the Selective Phase model suggests that the NPC central channel acts as a hydrogel that could be disrupted and reformed to allow passage of large molecules [68,69]. The Virtual Gating/Polymer Brush model indicates that peripheral entities of the NPC selectively signal the central channel to open its gate [70,71,72]. Such proposed models may not be mutually exclusive and hybrid models have also been proposed. For example, the Forest model proposes that there are two distinct zones within the central channel for passage of molecules; active transport occurs through the center and passive diffusion through the periphery of the central channel [73]. 

Because baculovirus nucleocapsids are among the largest cargos that cross the NPC, studies on nuclear import of baculovirus might provide important information that can be used to test the proposed models for NPC translocation. For example, the electron microscopy studies presented above demonstrate that the NPC is extremely flexible and must undergo a large scale of rearrangement to allow such a large nucleocapsid to occupy its central channel. As illustrated in Figure 4C, under conditions where transport across the NPC is not occurring, cytoplasmic filaments bend inward into the NPC central channel [66], while phenylalanine-glycine (FG) repeat domains of nucleoporins within this channel are natively unfolded and dispersed throughout this channel creating the typical electron‑dense appearance of the center of the NPC in electron micrographs of NPC cross-sections (Figure 4A). This ‘closed state’ does not allow the passage of large cargoes. The electron micrographs of AcMNPV nucleocapsids caught in the middle of the NPC (Figure 4B) indicate that the FG nucleoporins retract completely from the central channel, like an elevator door- or an iris-mechanism [74], leaving the 50‑nm in diameter NPC central channel completely open. It is possible that such a mechanism is only for large cargo, like the AcMNPV nucleocapsid, while other models may work only for small cargos. 

Although the electron microscopy and electron tomography of AcMNPV nucleocapsid nuclear import are highly suggestive, more studies are necessary to verify the re-localization of nucleoporins within the NPC central channel to allow for nucleocapsid transit through the NPC. In addition, proposed models should explain how the nucleocapsid could traverse an NPC without compromising the selective barrier of the NPC. The resulting ‘empty’ gap detected by electron tomography surrounding the nucleocapsid while it is in the middle of the NPC could provide an opening passageway for other molecules or proteins, resulting in an influx of theses into the nucleus. Immuno‑gold electron microscopy of nucleoporins may be able to determine whether NPC proteins (in particular FG domains) are located in this gap obstructing the transport of other cargoes, or even whether viral proteins associated with the nucleocapsid may fill this gap. 

## 5. Conclusions

The family *Baculoviridae* has evolved two very distinct mechanisms to deliver their viral genomes into the nucleus: (1) ejection of the nucleic acid through the NPC leaving an intact empty (devoid of DNA) capsid at the cytoplasmic side of the NPC, and (2) translocation of the intact DNA-containing nucleocapsid into the nucleus through the NPC, followed by the release of the viral genome in the nucleus. The type of mechanism used depends on the type of baculovirus genus, with Betabaculoviruses (GVs) using the first mechanism, and Alphabaculoviruses (NPVs) the second one. 

Studies of the nuclear import of baculovirus have been very descriptive and relied mostly in electron microscopy observations. Thus, cellular and viral proteins that mediate this process remain unidentified. The second type of mechanism triggers an opening of the NPC central channel. Thus, the unique large, rod-shaped nucleocapsid of baculovirus is an ideal experimental model to gain molecular details of the mechanism of nuclear transport. Studies of the nuclear import of baculovirus are certain to provide exciting new insights into how the NPC works, and are likely to lead to new procedures and conditions that will optimize the use of baculovirus in biomedical research and as an efficient insecticide.
